# “Not too far to walk”: the influence of distance on place of delivery in a western Kenya health demographic surveillance system

**DOI:** 10.1186/1472-6963-14-212

**Published:** 2014-05-10

**Authors:** Emily Mwaliko, Raymond Downing, Wendy O’Meara, Dinah Chelagat, Andrew Obala, Timothy Downing, Chrispinus Simiyu, David Odhiambo, Paul Ayuo, Diana Menya, Barasa Khwa-Otsyula

**Affiliations:** 1 College of Health Sciences, School of Medicine, Department of Reproductive Health, Moi University, P. O. Box 4606, Eldoret, Kenya; 2 College of Health Sciences, School of Medicine, Department of Family Medicine, Moi University, P.O. Box 4606, Eldoret, Kenya; 3 Duke University School of Medicine and Duke Global Health, Institute, Durham, NC, USA; 4 College of Health Sciences, School of Nursing, Moi University, P.O. Box 4606, Eldoret, Kenya; 5 College of Health Sciences, School of Medicine, Department of Microbiology, Moi University, P.O. Box 4606, Eldoret, Kenya; 6 USDA Forest Service, Santa Fe National Forest, 11 Forest Lane, Santa Fe, NM 87508, USA; 7 College of Health Sciences, School of Medicine, Department of Medicine, Moi University, P.O. Box 4606, Eldoret, Kenya; 8 College of Health Sciences, School of Medicine, Moi University HDSS Program Manager, P.O BOX 4606, Eldoret, Kenya; 9 College of Health Sciences, School of Public Health, Moi University, P.O. Box 4606, Eldoret, Kenya; 10 College of Health Sciences, School of Medicine, Department of Surgery, Moi University, P.O. Box 4606, Eldoret, Kenya

**Keywords:** Global positioning system, Demographic and surveillance system, Maternal health services, Emergency obstetric care, Hotspot analysis, Home/facility births

## Abstract

**Background:**

Maternal health service coverage in Kenya remains low, especially in rural areas where 63% of women deliver at home, mainly because health facilities are too far away and/or they lack transport. The objectives of the present study were to (1) determine the association between the place of delivery and the distance of a household from the nearest health facility and (2) study the demographic characteristics of households with a delivery within a demographic surveillance system (DSS).

**Methods:**

Census sampling was conducted for 13,333 households in the Webuye health and demographic surveillance system area in 2008–2009. Information was collected on deliveries that had occurred during the previous 12 months. Digital coordinates of households and sentinel locations such as health facilities were collected. Data were analyzed using STATA version 11. The Euclidean distance from households to health facilities was calculated using WinGRASS version 6.4. Hotspot analysis was conducted in ArcGIS to detect clustering of delivery facilities. Unadjusted and adjusted odds ratios were estimated using logistic regression models. P-values less than 0.05 were considered significant.

**Results:**

Of the 13,333 households in the study area, 3255 (24%) reported a birth, with 77% of deliveries being at home. The percentage of home deliveries increased from 30% to 80% of women living within 2km from a health facility. Beyond 2km, distance had no effect on place of delivery (OR 1.29, CI 1.06–1.57, p = 0.011). Heads of households where women delivered at home were less likely to be employed (OR 0.598, CI 0.43–0.82, p = 0.002), and were less likely to have secondary education (OR 0.50, CI 0.41–0.61, p < 0.0001). Hotspot analysis showed households having facility deliveries were clustered around facilities offering comprehensive emergency obstetric care services.

**Conclusion:**

Households where the nearest facility was offering emergency obstetric care were more likely to have a facility delivery, but only if the facility was within 2km of the home. Beyond the 2-km threshold, households were equally as likely to have home and facility deliveries. There is need for further research on other factors that affect the choice of place of delivery, and their relationships with maternal mortality.

## Background

Kenya has a high maternal mortality ratio of 488 per 100,000 live births. This has not changed much from the last Kenya Demographic and Health Survey [1] conducted in 2003. The fifth millennium development goal is to reduce maternal mortality to 147 per 100,000 live births by 2015; however, the coverage of maternal health services remains low. It is commonly accepted that mothers who deliver in a health facility have better outcomes than those who deliver at home [2]. Yet in Kenya, 56% of women delivered at home in 2009. In western Kenya, the proportion of home deliveries is even higher at 73% [1].

Several studies in East Africa have investigated reasons for the prevalence of home deliveries [2-6]. Although the reasons vary, some are commonly cited: women living in a rural area, having little education, having low socio-economic status, being a long way from a health facility, having had a previous delivery at home, having had little or no antenatal care, and being multiparous. One review study [7] grouped determinants of the place of delivery under four themes in an adapted framework: 1) socio-cultural factors, 2) perceived benefit of/need for skilled attendance, 3) economic accessibility and 4) physical accessibility. These domains demand distinct approaches to overcome the barriers suggested by each. Therefore, understanding the relative contributions of these factors is important.

A recent geographical information study [8] in Zambia focused on physical accessibility and linked national household data with national facility data to look at correlations between place of delivery (home or facility) and both distance of the mother from a facility and the level of care offered at the facility. As the distance from the closest health facility increased, the odds of facility delivery decreased—a finding that is in tandem with the findings of other studies. However, the study [8] also found that the odds of health-facility birth were higher if the closest facility offered comprehensive care. For instance, a woman who lived a short distance from a facility offering a high level of health care was more likely to deliver at the facility than a woman who lived a short distance from a facility offering a lower level of health care.

These results suggest interaction between physical access and perceived benefit of care in the decision to deliver at a facility. In a Zambian study [8], half of all births were to mothers living more than 25 km from a facility offering at least basic emergency obstetric care. Here we report a study carried out in an area where physical access to delivery facilities is much closer than 25 km for the entire population. The objectives were to (1) determine the association between the place of delivery and the distance of a household from the nearest health facility and (2) study the demographic characteristics of households with a delivery within a demographic surveillance system (DSS).

## Methods

### Study setting

This study was conducted using data from the Webuye Health and Demographic Surveillance System. The DSS is located in Bungoma County of the former Western Province, and is approximately 400 km west of Nairobi. The study site is an area approximately 24km from north to south, and 2–6km east to west. The total area is 130km^2^ with a population of about 77,000 people living in 13,333 households. About 61% of the population lives below the poverty line, and social amenities like water and electricity are not readily available to the majority. There is one 100-bed mission hospital within the study area and one 200-bed district hospital adjacent to the study area, both offering comprehensive emergency obstetric care. There are also several dispensaries, staffed by nurses and offering outpatient care, and one health center offering 24-hour delivery services but without the capacity to perform cesarean sections.

### Study design

This was a cross-sectional community-based study using data obtained from the Webuye health and demographic surveillance system (HDSS) database between 2008 and 2009. Each household was geo-referenced using the Global Positioning System (GPS).

### Study population and sampling technique

The study included all households within the Webuye HDSS that were registered during the baseline and subsequent censuses, and had reported at least one birth within one year preceding the census.

### Study instruments and data collection

Data were collected via structured interviews with the assistance of trained field assistants. The contents of the interview schedules were adapted from the standard INDEPTH [9] questionnaires developed by various HDSS sites. Various stakeholders in the surveillance activities met to discuss key contents of the questionnaires, modified some of the existing questions and designed new questions to reflect the local situation. The questionnaires were further refined after a pilot study prior to the distribution of the final versions to the field staff. All household data were collected via interviews with the head of the household and from GPS coordinates of each household; therefore, we present data of the women’s immediate environment (household) rather than her individual characteristics (Table 1).

**Table 1:** Descriptive statistics

**Household delivery data**		
**Percent of homes with at least one birth at facility**		30.8%
**Percent of households with at least one birth at home**		77%
**Percent of households with head=male**		90%
**Employment status of head**		
	Unemployed	13.4%
	Employed	20.5%
	Self-employed	65.4%
	Head is farmer	42.8%
	Head owns business	9.7%
	Head is salaried	15.2%
	Head is skilled laborer	12.3%
	Head is casual laborer	18.7%
**Education of head**		
	No education	1.36%
	Pre-primary	0.22%
	Primary	58.68%
	Secondary	33.20%
	Technical/Vocational	3.12%
	Higher	2.96%
	Informal	0.46%
**Mean household size**		6.5 people (SD=2.9)
	Mean people per room	3.1 (SD=1.55)
	Mean number of rooms	2.3
	Mean acres of land owned	1.77
	Mean distance to facility	2.4 km (SD=1.13)
	Mean distance to road	3.11 km (SD=1.55)
	Nearest facility is hospital (percent)	33.8%
	Nearest facility is health center (percent)	12.9%
	Nearest facility is dispensary (percent)	53.3%

The household questionnaire gathered basic information from the head of the household on usual members of and visitors to the household, including age, sex, education level, and relationship to the head of the household. Information was also collected on deliveries that had occurred during the previous 12 months and socio- economic characteristics of the household’s dwelling unit, such as the source of water, property ownership and possession of mosquito nets. Digital coordinates were also collected for the households and sentinel locations such as health facilities using GPS units.

### Data management and analysis

Completed questionnaires were first checked in the field by the field supervisors for completeness. The questionnaires were then sent to the field office where data- quality checkers reviewed the forms for completeness, logic and consistency. The incorrectly filled questionnaires were returned to the respective field interviewers for correction. The correctly filled questionnaires were passed over to the data entry clerks for data entry. After data entry, questionnaires with questionable records identified through automated internal consistency checks were sent back to the field interviewers for verification and correction. The data were stored in a Mysql database (Mysqlab Inc., Uppsala, Sweden).

All data were organized and analyzed using STATA version 11 (StataCorp, 2011). Distance from households to health facilities was calculated as Euclidean distance using WinGRASS version 6.4. Hotspot analysis was done in ArcGIS using Hotspot Analysis within the Spatial Statistics toolset to detect clustering of facility deliveries. The demographic and baseline outcomes were recapitulated using descriptive summary measures expressed as the sum, mean, median and standard deviation for continuous variables and percentages for categorical variables. Unadjusted and adjusted odds ratios were estimated using logistic regression models. P-values less than 0.05 were considered significant. Three multivariate models using different covariates to describe access to facilities were explored. Model 1 included distance to any facility as a continuous measure and the type of nearest facility, Model 2 categorized distance to the nearest facility using a threshold, and Model 3 categorized distance to the hospital using a threshold. The best model was selected using Akaike Information Criterion (AIC, Additional file 1: Table S1).

### Ethical considerations

The study received ethical clearance from the joint Institutional Research and Ethics Committee of Moi University and Moi Teaching and Referral Hospital. Clearance certificate number IREC/2008/05 (for the period 24th April 2008 to March 2009) was obtained before commencement of the data collection.

## Results

### Demographics

During the year prior to commencement of this study (beginning January 2009), 3255 registered households within the DSS study area had reported a birth, out of which 139 households had more than one birth. The reasons were some households have polygamous heads, in some the sons marry while still living within the parents’ home (the household). The majority of households (77%) had a home delivery, compared with 30.8% who had a health-facility delivery (Table 1). As there were some households with more than one birth during the study period, the total percentage of births at home plus those in a facility is greater than 100%. Farming was the most common occupation of household heads (42.8%), followed by casual labor (18.7%) and salaried work (15.2%). Most household heads had only primary education or less (60.26%). The mean number of acres owned per household was 1.77. The mean distance from a health facility was 2.4 km.

The primary outcome was any delivery at home in the last year. Unadjusted odds ratios are presented in Table 2 and adjusted odds ratios are reported in Table 3. The employment status of the head was included in the multivariable model, but the type of employment was not because these variables are highly correlated. The relationship between delivery at home and distance to a facility is biphasic and therefore divided into categories after inspection of the empiric relationships. Multivariate analysis shows that a woman who delivers at home is less likely to come from a household where the head has secondary rather than primary education (OR 0.49, p < 0.0001) and is less likely to be employed (OR 0.58, p = 0.001) than a woman who delivers at a health facility. Compared with facility delivery, delivery at home was also associated with more people per room in each household (higher household crowding) (OR 1.16, p = 0.001).

**Table 2:** Bivariate analysis of household covariates and home delivery

		**Bivariate analysis**			
**Variable**		**N**	**Unadjusted OR**	**P**	**Value 95% CI**
**Age of head**		3103	0.99	0.876	0.99 - 1.00
**Head of male**		3141	0.94	0.663	0.71 - 1.24
**Education of head**		3142			
	Primary or below		1	REF	
	Secondary or above		0.41	0.0001	0.35 - 0.49
**Employment of head [ii]**		3142			
	Formally employed		0.43	0.0001	0.32 - 0.57
	Self employed		1.06	0.66	0.82 - 1.37
	Unemployed		1	REF	
**Employment type of head [iii]**		3169			
	Owns business		0.67	0.006	0.50 - 0.89
	Salaried		0.37	0.0001	0.30 - 0.46
	Skilled laborer		0.99	0.95	0.75 - 1.31
	Casual laborer		1.16	0.254	0.90 - 1.48
	Farmer		1	REF	
**Household size**		3115	1.06	0.0001	1.03 - 1.10
**People per room**		3068	1.26	0.0001	1.18 - 1.34
**Acres of land owned**		3142	0.99	0.39	0.98 - 1.01
**Nearest facility [iv]**		2855			
	Hospital		0.55	0.0001	0.46 - 0.66
	Health Center		1.01	0.953	0.75 - 1.35
	Dispensary		1	REF	
**Distance in kilometres**		2855			
	To any facility (kms)		1.09	0.0311	1.01 - 1.18
	To District Hospital (kms)		1.1	0.0001	1.08 - 1.13
	To Road (kms)		1.46	0.011	1.09 - 1.95
**Distance categories**		2855			
	<2km from any facility		1	REF	
	>2 km from any facility		1.21	0.035	1.01 - 1.46
	<4km from District Hospital		1	REF	
	>4km from District hospital		2.32	0.0001	1.90 - 2.83

**Table 3:** Multivariate analysis

**Variable**		**OR**	**P value**	**95% CI**
**Age of head**		0.99	0.050	0.98 - 1.00
**Education of head**				
	Primary or below		1	REF
	Secondary or above	0.49	<0.0001	0.40 - 0.60
**Employment of head**				
	Formally employed	0.58	0.001	0.42 - 0.81
	Self employed	1.10	0.520	0.82 - 1.47
**Household size**		1.03	0.185	0.98 - 1.08
**People per room**		1.16	0.001	1.06 - 1.26
**Acres of land owned**		1.01	0.531	0.99 - 1.03
**Distance in kilometres**				
	To road (kms)	1.14	0.403	0.84 - 1.56
**Distance categories**				
				
	<4km from District Hospital		1.00	REF
	>4km from District hospital	2.07	<0.0001	1.66 - 2.57

### Home delivery and geographic access to care

**Figure 1:**
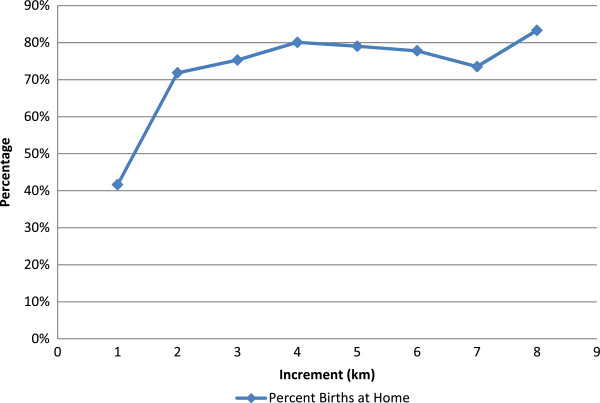
Percent births at home.

Both the distance to a facility and the type of services offered at the nearest facility were correlated with delivering at home. The average distance from a household to the nearest facility of any type is 2.4 km. The proportion of households where women delivered at home increased with distance from a health facility. Figure 1 shows that home deliveries increase sharply from 30% to over 70% at a distance of about 2 km away from a health center or hospital. However, if a woman lives more than about 2 km from a facility, regardless of the services offered, she is as likely to deliver at home as if she lives 4, 6 or 8 km away.

Women in households that are closer to a hospital than a dispensary were half as likely to have a home delivery (Table 2), even when correcting for education and employment (Additional file 1: Table S1, Model 1). Distance to the hospital was strongly negatively correlated with delivering at home; the odds of delivering at home was doubled for women who lived more than 4 kilometers from a hospital (adjusted OR 2.07, p < 0.0001. Table 3), even after adjusting for education, employment and distance to a road (OR 2.07, CI 1.08–1.60, p = 0.011; Table 3).

**Figure 2:**
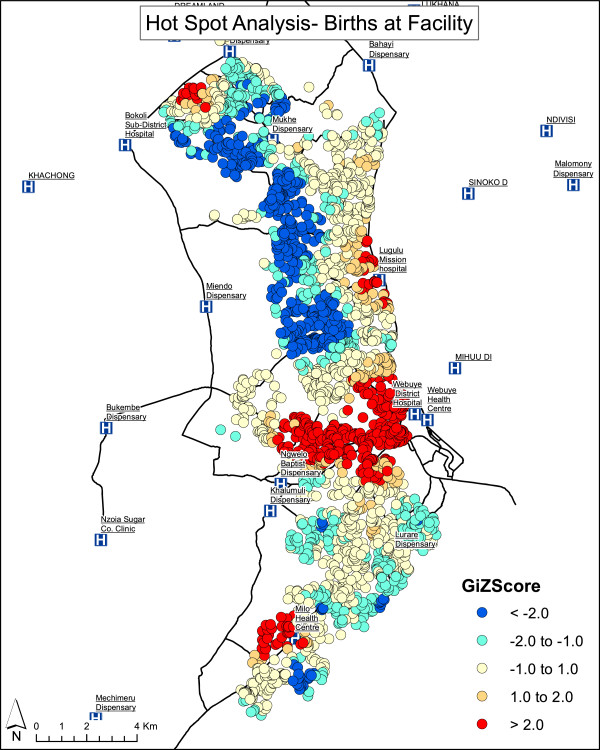
Hot spot analysis- births at facility.

Distance to a road was included to correct for the possible difference between Euclidean distance and actual travel time or access to transportation. Distance to a road was significant in the univariate analysis (p = 0.011), but not in the multivariate analysis (p = 0.403). GPS coordinates were missing for 287 households (9%). There was no difference in terms of employment of the head of the household, age of the head of the household, household size or education of the head of the household between households with and without GPS coordinates. Hotspot analysis showed that households choosing facility deliveries are significantly clustered around the two major hospitals offering emergency obstetric care and cesarean sections (Figure 2). Smaller but significant clusters were observed around one health center in the southern part of the study area. No significant clusters of facility delivery were identified around dispensaries. Therefore, we analyzed the location of delivery with reference to the type of facility. Dispensaries generally offer delivery services only on weekdays during the day; health centers offer 24-hour delivery services, but no operative emergency obstetric care such as cesarean sections; hospitals offer 24-hour delivery services as well as emergency obstetric care. Those families for whom a dispensary was the nearest facility were less likely to deliver in a facility. Those whose nearest facility was a health center or hospital (Figure 1) were more likely to deliver in a facility, but only if they lived within 2 km. This study did not analyze the relationship between the births and the time of day.

## Discussion

This study confirms what has been previously described, that women who deliver at home are more likely to be of lower socio-economic status [5,6] and are more likely to live far from a maternity facility [3-6]. However, we found a threshold distance of about 2 km, beyond which distance ceased to be a major determinant of home delivery. This distance was substantially less than that found by previous studies. In Zambia, Gabrysch et al. [8] found that the percentage of women delivering in a facility began falling off at a distance of 5km from a facility. Two other studies [4,5] described a similar 5-km cut-off, but it is unclear whether this distance was determined from analysis of distance as a continuous variable, or whether the cut-off was simply a pre-chosen categorical variable. In addition, we found that beyond about 2 km, the percentage of women delivering in a facility did not continue to decline with distance (Figure 1). Clearly there are many factors affecting a woman’s decision to deliver at home, and this study did not investigate factors other than distance from a facility and household socioeconomic status. Yet, because previous studies have emphasized the distance factor in their titles—“Too Far To Walk” [10] and “Still Too Far To Walk” [7]—our contribution questions the pivotal role of distance suggested in these studies, at least for our population. Although we cannot propose from our findings reasons why women deliver at home, our very high home delivery rate (73%), even for women who live relatively close (<5 km) to a hospital or health facility, suggests that there are other factors we have not yet uncovered. A qualitative study in Laos [11] pointed strongly to cultural reasons why women there deliver at home, and also highlighted discomfort some women felt with impersonal, institutional deliveries, and the perception of poor quality of care in hospitals. A study in Malawi and Zambia [12] looked at geographic access and neonatal outcomes while another in Ghana [13] looked at quality of care. Neither study concluded that better geographic access was associated with lower neonatal mortality. In an analysis based on Kenya Demographic and Health Survey considering place of delivery [14], the majority of the women cited distance as the reason for delivering at home. However those living less than 2 km from a health facility cited cost as the main reason for delivering at home. These studies indicate that there are other factors that determine why women don’t utilize health facilities for delivery. These factors need to be investigated in our study area, especially as facility deliveries continue to be promoted as a means of reducing maternal mortality. A recent review of such strategies [15] included as a centerpiece health-center-based deliveries for all women. It, along with a companion study [16], emphasized the need for political and financial commitment at the district level to achieve this goal—a commitment they felt was lacking. Gabrysch et al. [17] analyzed health system output indicators in high mortality and low mortality countries, and concluded that these need to be revised and contextualized. Their recommendation is that data needs to be disaggregated to the subnational level to explain inequalities and also to help at the district level planning.

One of the limitations of this study was time and budget. It was not practical to include observations or analysis of quality of care provision in health facilities. The data was from the DSS database and only heads of households were interviewed, not each mother. They were not specifically asked whether the mothers were resident in the study site for their deliveries. Information from household heads, being mostly male and not present at the deliveries, limits the current study to questions of distance. Mother’s preference of delivery site, reasons for her choice, and quality of care received/perceived were not investigated.

Clearly, if cultural factors and poor quality of facility care are confirmed in further studies to be principle reasons for home deliveries, the implications relate more to community education and facility improvement rather than simply building more health facilities closer to where people live.

## Conclusion

This study shows that distance to a health facility is not a factor affecting the decision of place of delivery. This is contrary to the findings of many studies that showed distance to be a major factor. There is need for further research (both qualitative and quantitative) on other factors that affect the choice of place of delivery, and possible relationships between the research results and maternal mortality within the same community should be explored.

## Abbreviations

HDSS: Health and Demographic Surveillance System; GPS: Global Positioning System; IREC: Institutional Research and Ethics Committee; OR: Odds ratio; CI: Confidence interval; Household: A group of persons who live, eat (use the same cooking fire) and sleep within the same housekeeping arrangement.

## Competing interest

The authors declare that they have no competing interests.

## Authors’ contributions

RD analyzed data and drafted the manuscript. WO contributed to data analysis and interpretation. EM, DC and AO participated in concept design and data analysis. CS contributed to the methodology and acquisition of data. TD was involved in GIS analysis and production of maps. PA, DO, DM and BO approved the manuscript for publication. All authors read and approved the final manuscript.

## Pre-publication history

The pre-publication history for this paper can be accessed here:

http://www.biomedcentral.com/1472-6963/14/212/prepub

## Supplementary Material

Additional file 1: Table S1 Comparing multivariate models of home delivery. Click here for file
