# Supplementary material for: “Not too far to walk”: the influence of distance on place of delivery in a western Kenya health demographic surveillance system
Source: BMC Health Serv Res. 2014 May 10;14:212. doi: 10.1186/1472-6963-14-212 (PMC4036729; doi:10.1186/1472-6963-14-212)
Supplement: Additional file 1: Table S1 — Comparing multivariate models of home delivery. [file 1472-6963-14-212-S1.pdf]

Table S1: Comparing multivariate models of home delivery

n=2696	MODEL 1			MODEL 2			MODEL 3		
**Variable**	**OR**	**P value**	**95% CI**	**OR**	**P value**	**95% CI**	**OR**	**P value**	**95% CI**
Age of Head	0.99	0.101	0.98 - 1.00	0.99	0.104	0.98 - 1.00	0.99	0.050	0.98 - 1.00
**Education of head**									
Primary or below	REF								
Secondary or above	0.50	<0.0001	0.41 - 0.61	0.50	<0.0001	0.41 - 0.61	0.49	<0.0001	0.40 - 0.60
**Employment of head**									
Formally employed	0.57	0.001	0.41 - 0.79	0.57	0.001	0.41 - 0.79	0.58	0.001	0.42 - 0.81
Self employed	1.07	0.650	0.80 - 1.43	1.07	0.664	0.80 - 1.43	1.10	0.520	0.82 - 1.47
Unemployed	1.00	REF		1.00	REF				
**Household size**	1.03	0.174	0.99 - 1.09	1.04	0.151	0.99 - 1.09	1.03	0.185	0.98 - 1.08
**People per room**	1.14	0.002	1.05 - 1.25	1.14	0.003	1.05 - 1.24	1.16	0.001	1.06 - 1.26
**Acres of land owned**	1.00	0.731	0.98 - 1.02	1.00	0.694	0.98 - 1.02	1.01	0.531	0.99 - 1.03
**Nearest facility**									
Hospital	0.59	<0.0001	0.48 - 0.73	0.60	<0.0001	0.49 - 0.73			
Health Center	0.99	0.951	0.72 - 1.36	0.97	0.840	0.70 - 1.33			
Dispensary	1.00	REF		1.00	REF				

**Distance in kilometers**												
To any facility (kms)	1.14	0.002	1.05	- 1.25								
To Road (kms)	1.20	0.274	0.87	- 1.65	1.26	0.158	0.91	- 1.74	1.14	0.403	0.84	- 1.56
**Distance categories**												
<2km from any facility					1.00	REF						
>2 km from any facility					1.32	0.006	1.08	- 1.60				
<4km from District Hospital									1.00	REF		
>4km from District hospital									2.07	<0.0001	1.66	- 2.57
AIC	*2725.868*				*2727.703*				*2712.782*			
BIC	*2796.662*				*2798.497*				*2771.777*			
